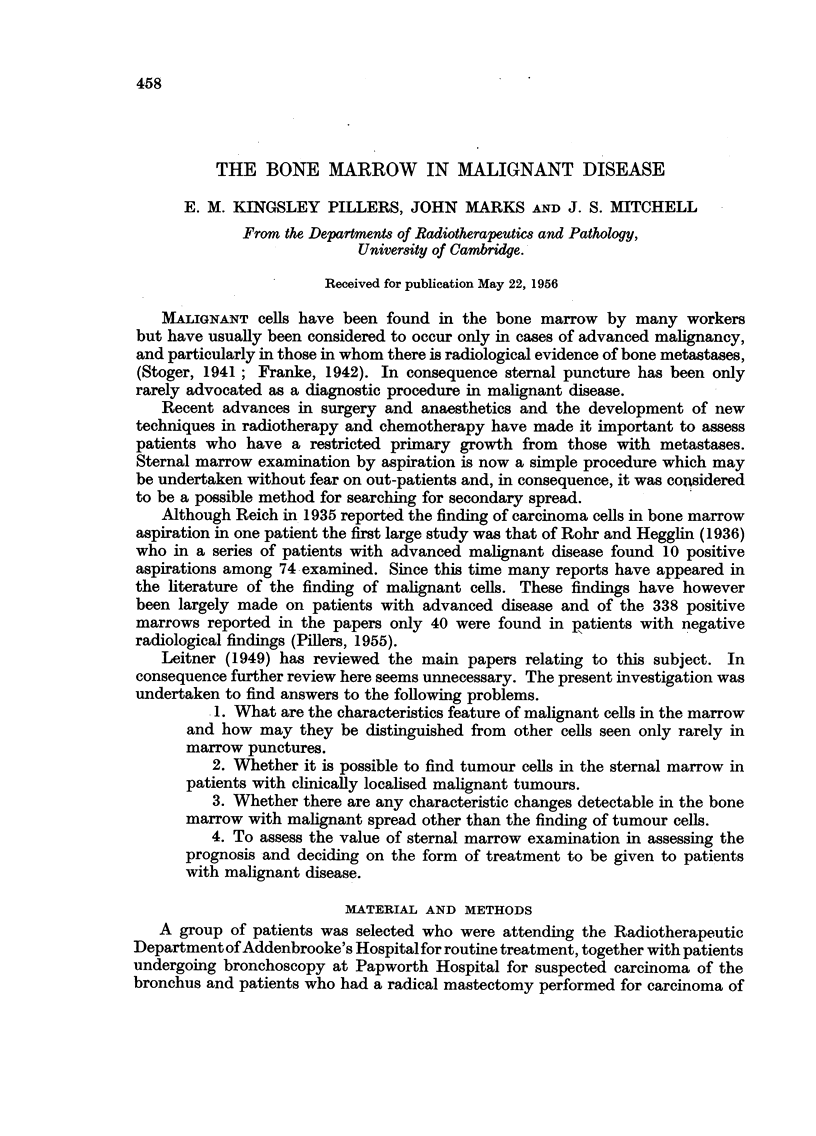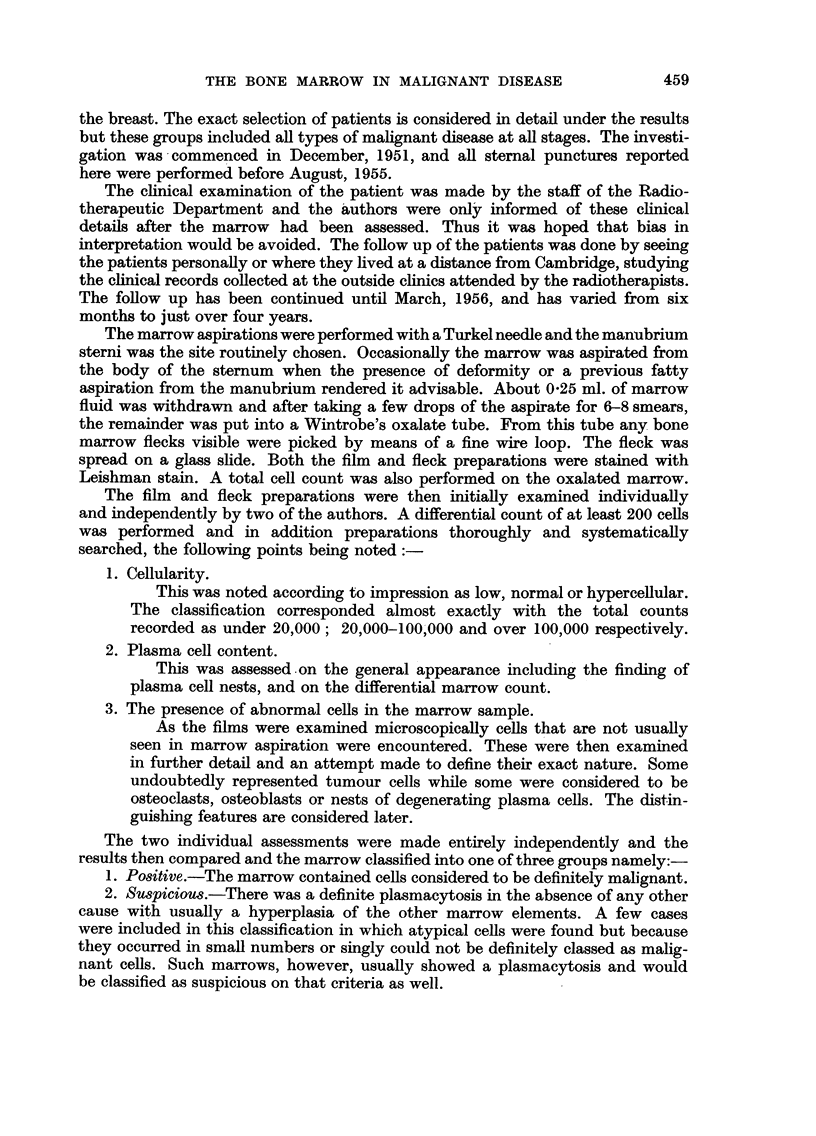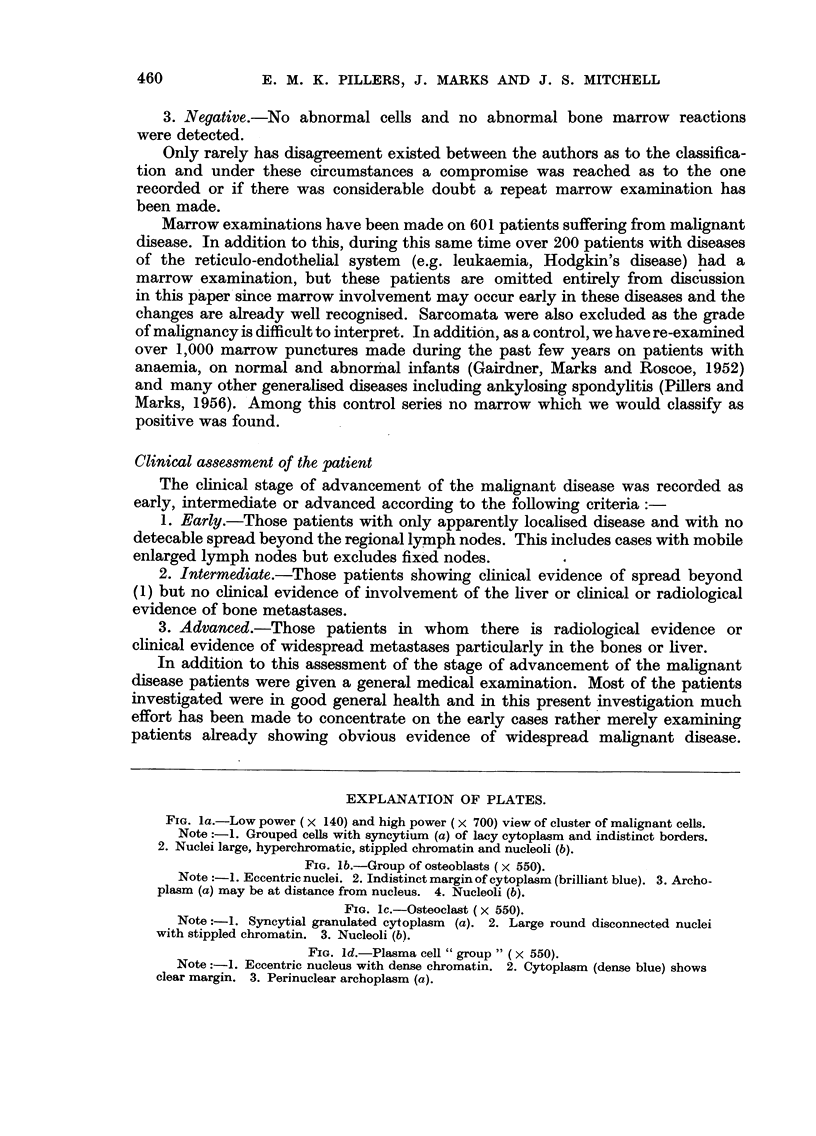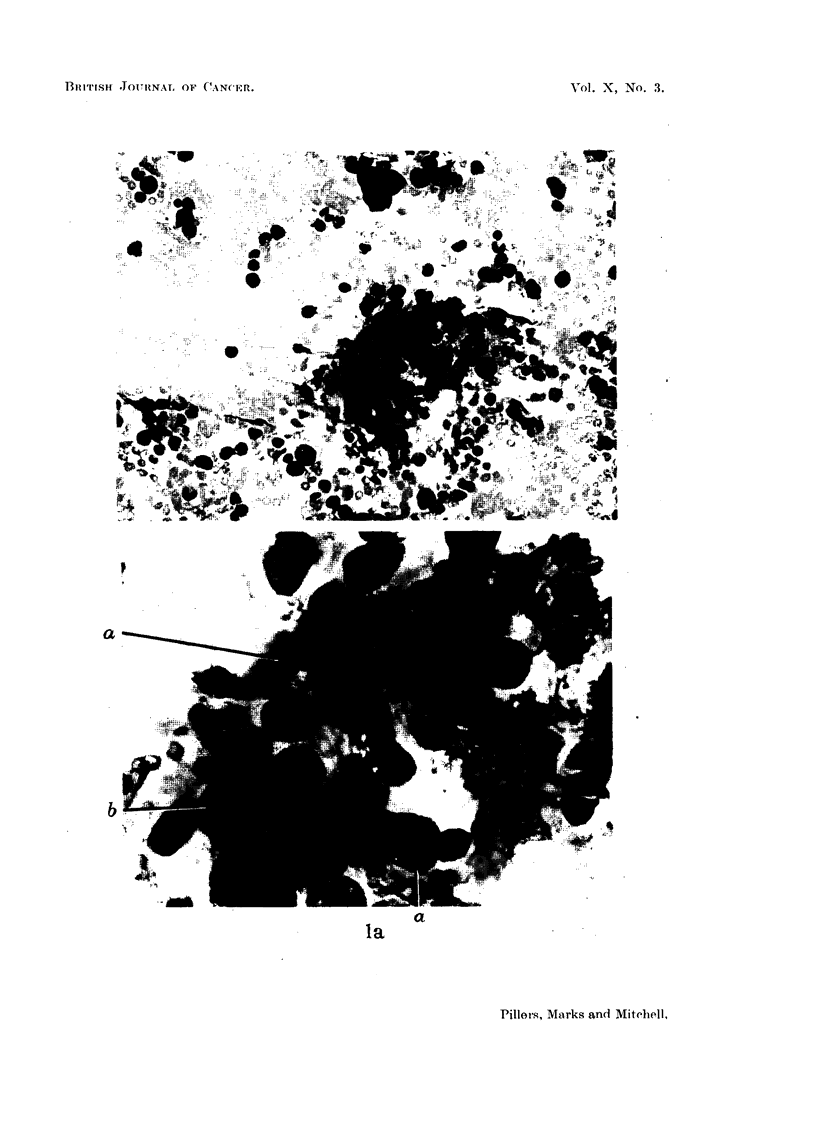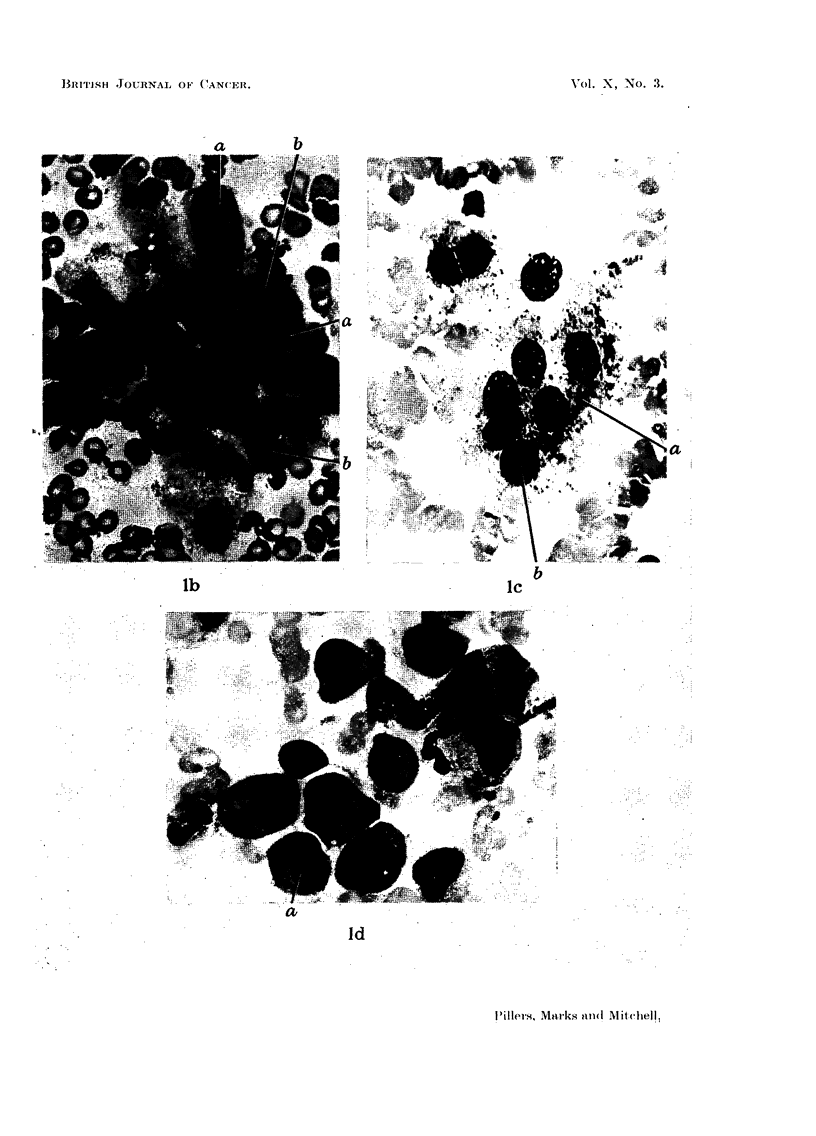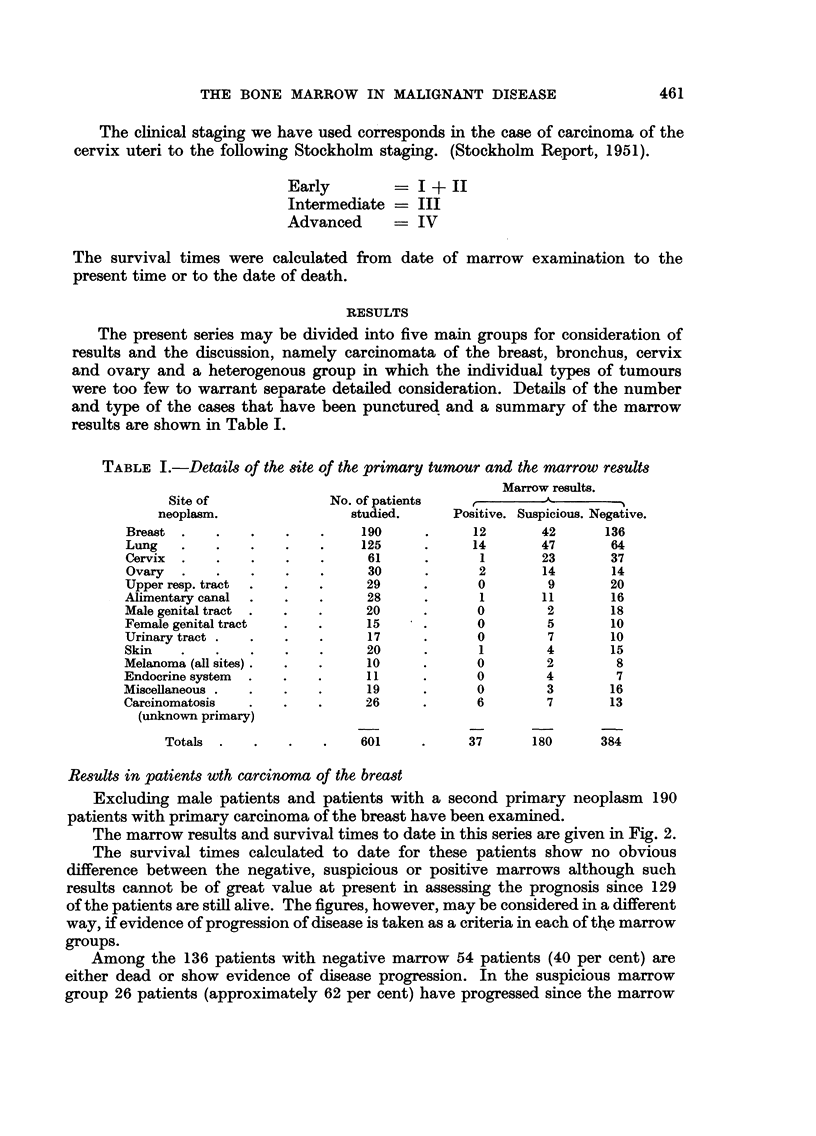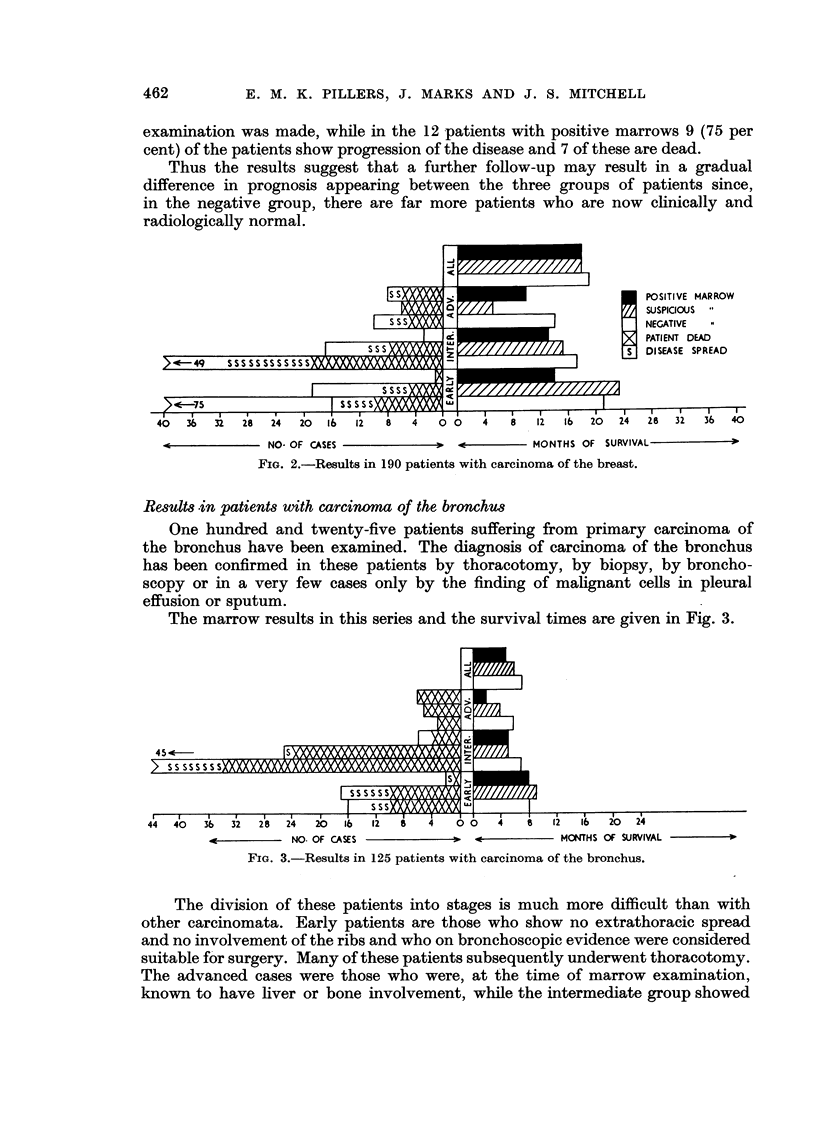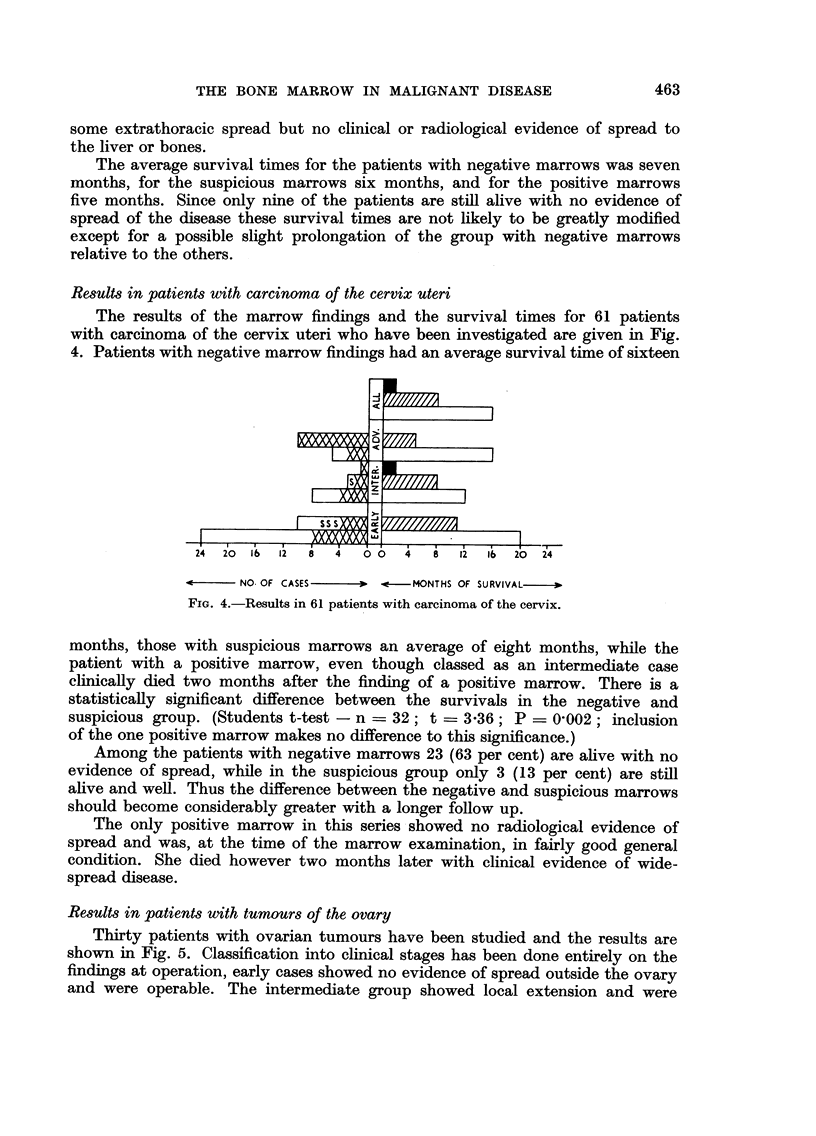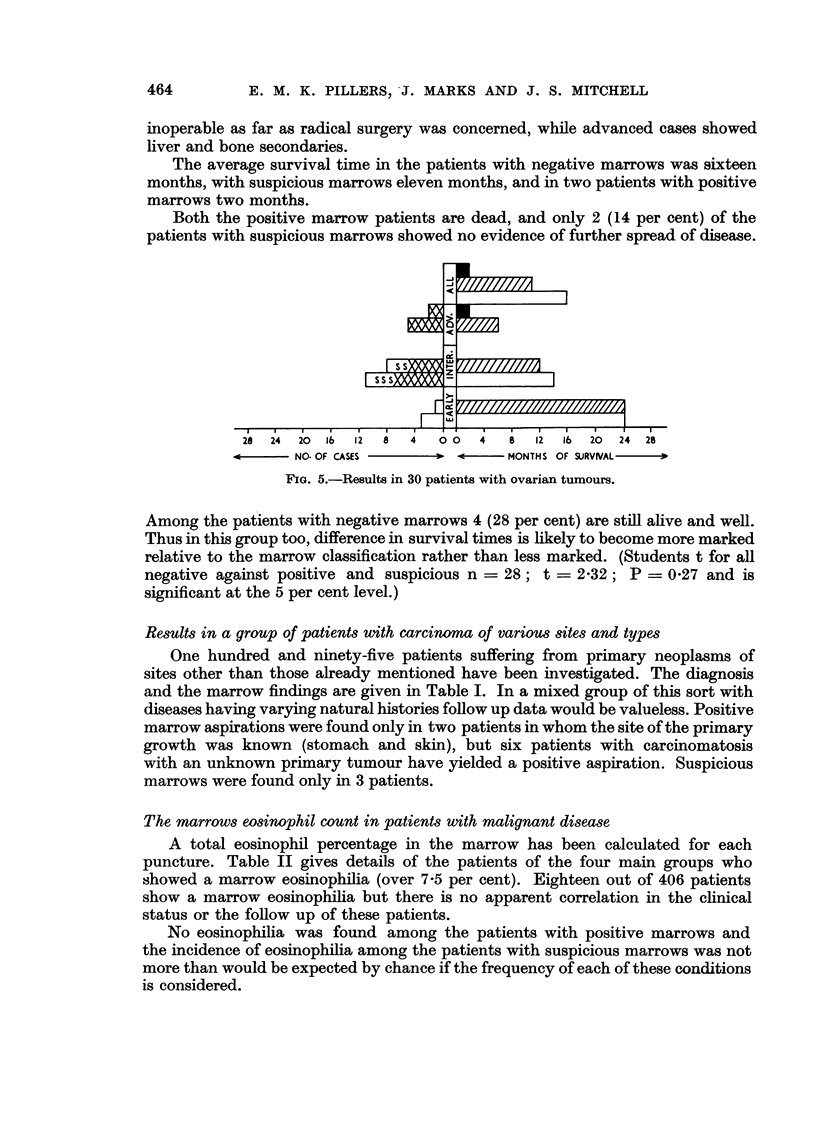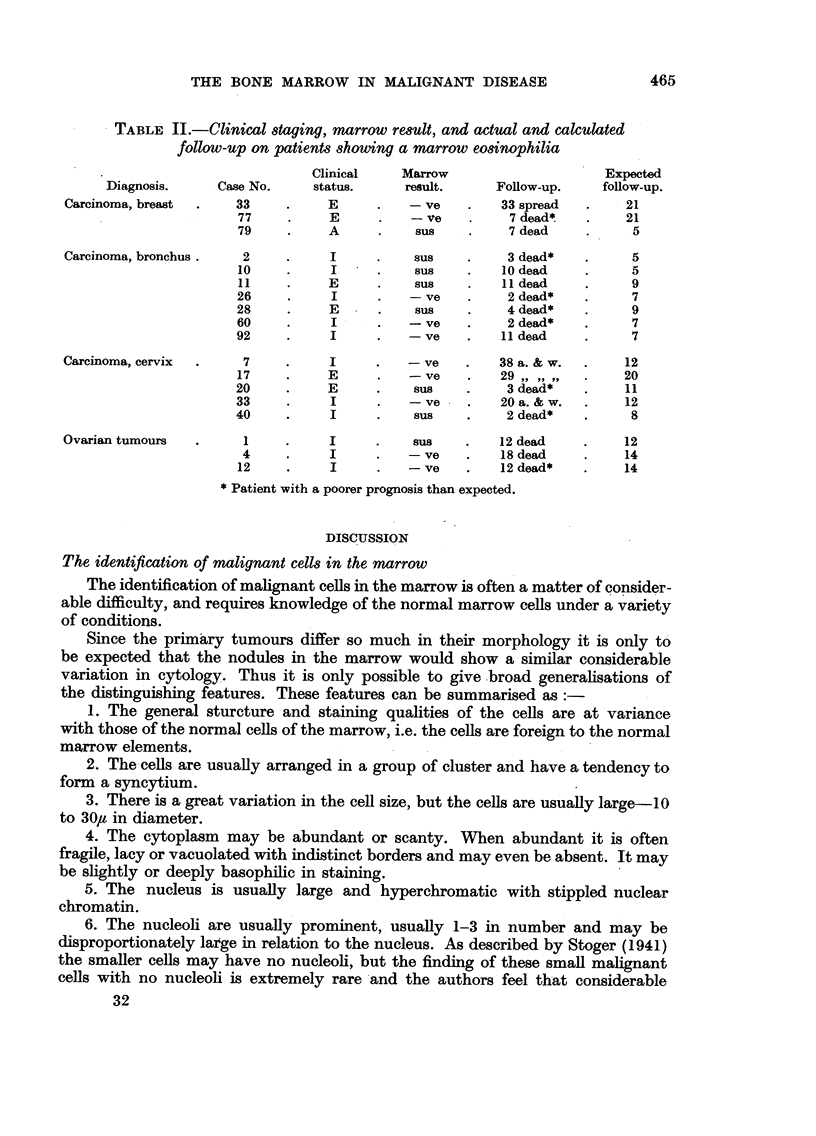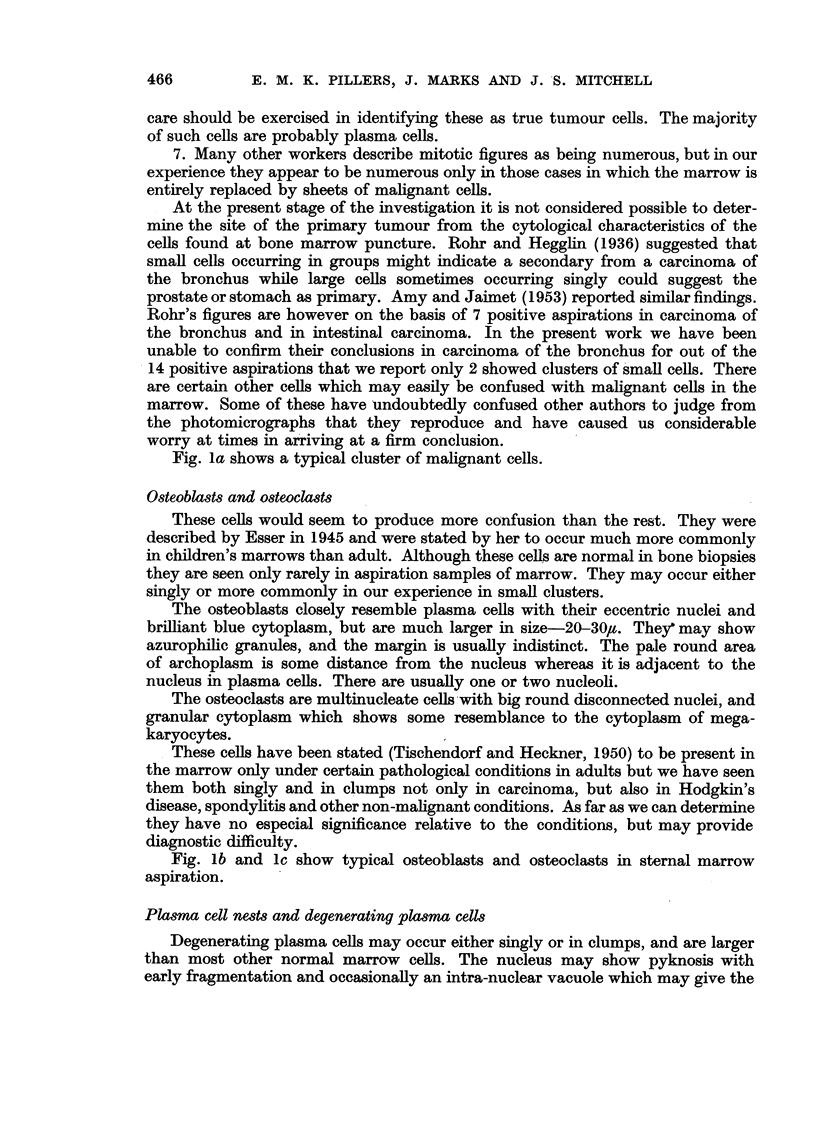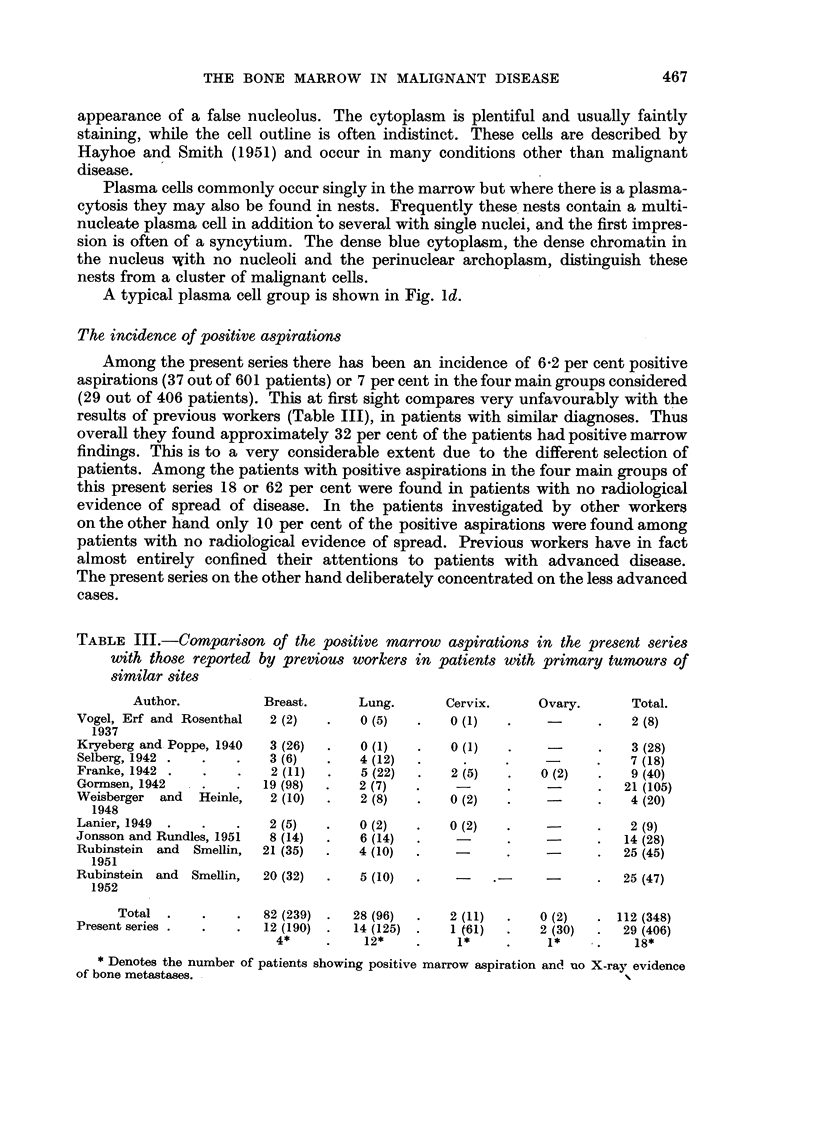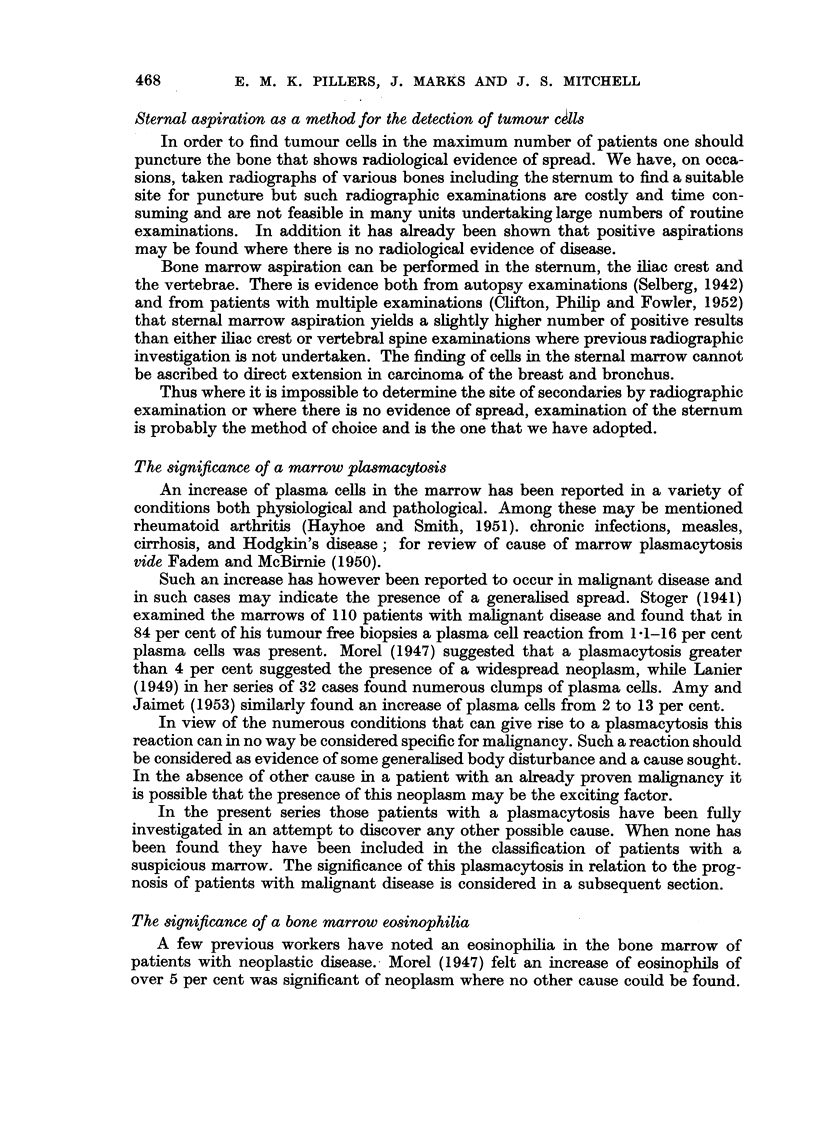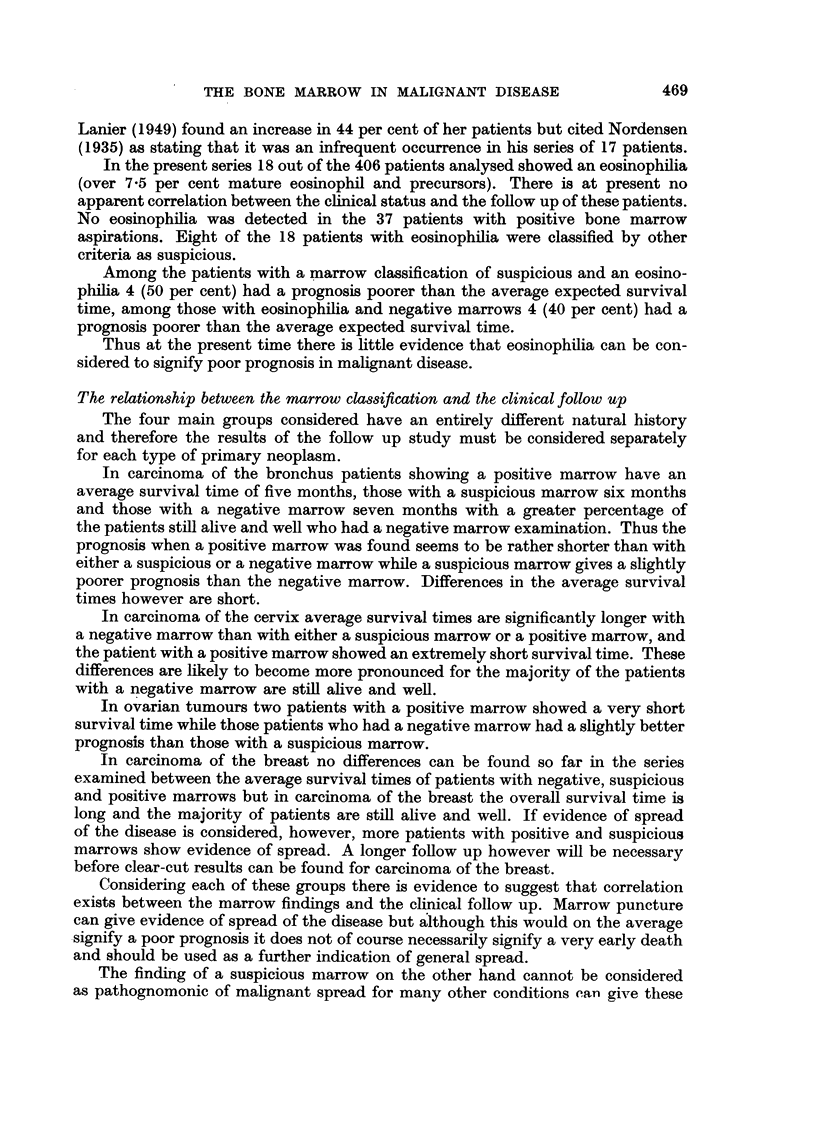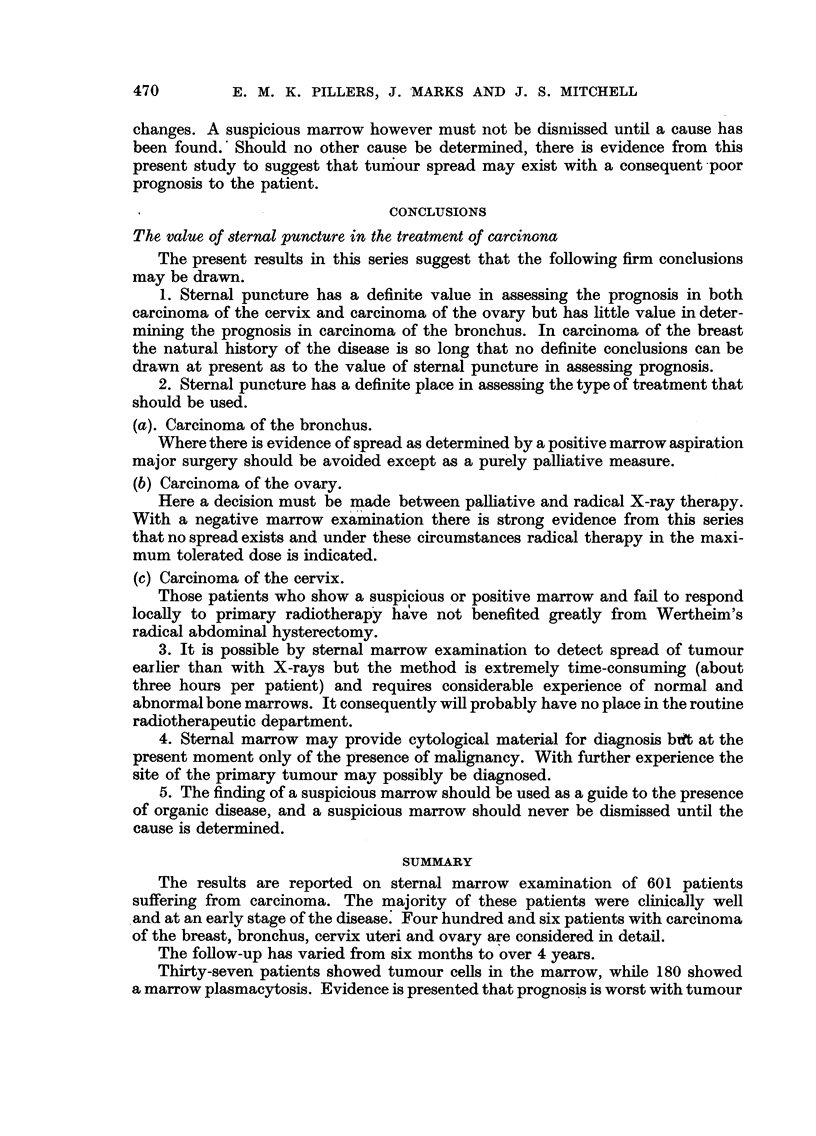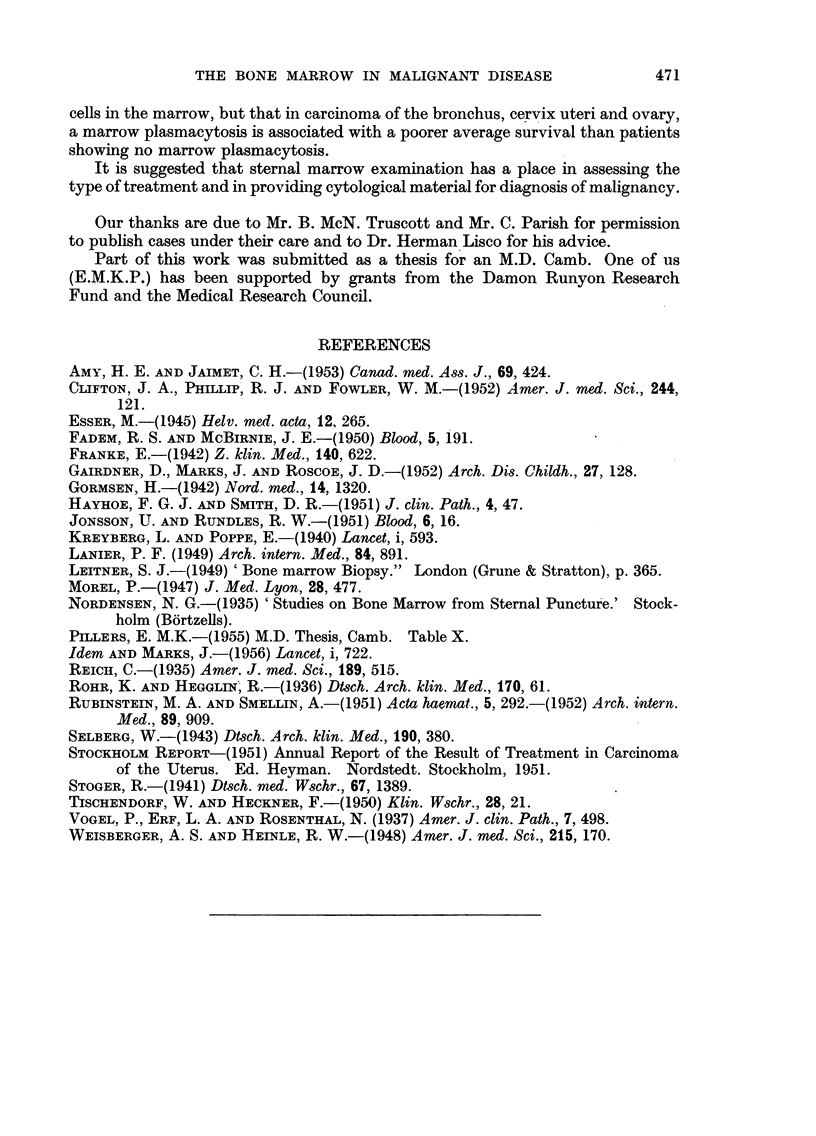# The Bone Marrow in Malignant Disease

**DOI:** 10.1038/bjc.1956.52

**Published:** 1956-09

**Authors:** E. M. Kingsley Pillers, John Marks, J. S. Mitchell

## Abstract

**Images:**


### THE BONE MARROW IN MALIGNANT DISEASE

E. M. KINGSLEY PILLERS, JOHN MARKS AND J. S. MITCHELL

*From the Departments of Radiotherapeutics and Pathology, University of Cambridge.*

Received for publication May 22, 1956

MALIGNANT cells have been found in the bone marrow by many workers but have usually been considered to occur only in cases of advanced malignancy, and particularly in those in whom there is radiological evidence of bone metastases, (Stoger, 1941 ; Franke, 1942). In consequence sternal puncture has been only rarely advocated as a diagnostic procedure in malignant disease.

Recent advances in surgery and anaesthetics and the development of new techniques in radiotherapy and chemotherapy have made it important to assess patients who have a restricted primary growth from those with metastases. Sternal marrow examination by aspiration is now a simple procedure which may be undertaken without fear on out-patients and, in consequence, it was considered to be a possible method for searching for secondary spread.

Although Reich in 1935 reported the finding of carcinoma cells in bone marrow aspiration in one patient the first large study was that of Rohr and Hegglin (1936) who in a series of patients with advanced malignant disease found 10 positive aspirations among 74 examined. Since this time many reports have appeared in the literature of the finding of malignant cells. These findings have however been largely made on patients with advanced disease and of the 338 positive marrows reported in the papers only 40 were found in patients with negative radiological findings (Pillers, 1955).

Leitner (1949) has reviewed the main papers relating to this subject. In consequence further review here seems unnecessary. The present investigation was undertaken to find answers to the following problems.

1. What are the characteristics feature of malignant cells in the marrow and how may they be distinguished from other cells seen only rarely in marrow punctures.
2. Whether it is possible to find tumour cells in the sternal marrow in patients with clinically localised malignant tumours.
3. Whether there are any characteristic changes detectable in the bone marrow with malignant spread other than the finding of tumour cells.
4. To assess the value of sternal marrow examination in assessing the prognosis and deciding on the form of treatment to be given to patients with malignant disease.

### MATERIAL AND METHODS

A group of patients was selected who were attending the Radiotherapeutic Department of Addenbrooke's Hospital for routine treatment, together with patients undergoing bronchoscopy at Papworth Hospital for suspected carcinoma of the bronchus and patients who had a radical mastectomy performed for carcinoma of the breast. The exact selection of patients is considered in detail under the results but these groups included all types of malignant disease at all stages. The investigation was commenced in December, 1951, and all sternal punctures reported here were performed before August, 1955.

The clinical examination of the patient was made by the staff of the Radiotherapeutic Department and the authors were only informed of these clinical details after the marrow had been assessed. Thus it was hoped that bias in interpretation would be avoided. The follow up of the patients was done by seeing the patients personally or where they lived at a distance from Cambridge, studying the clinical records collected at the outside clinics attended by the radiotherapists. The follow up has been continued until March, 1956, and has varied from six months to just over four years.

The marrow aspirations were performed with a Turkel needle and the manubrium sterni was the site routinely chosen. Occasionally the marrow was aspirated from the body of the sternum when the presence of deformity or a previous fatty aspiration from the manubrium rendered it advisable. About 0·25 ml. of marrow fluid was withdrawn and after taking a few drops of the aspirate for 6–8 smears, the remainder was put into a Wintrobe's oxalate tube. From this tube any bone marrow flecks visible were picked by means of a fine wire loop. The fleck was spread on a glass slide. Both the film and fleck preparations were stained with Leishman stain. A total cell count was also performed on the oxalated marrow.

The film and fleck preparations were then initially examined individually and independently by two of the authors. A differential count of at least 200 cells was performed and in addition preparations thoroughly and systematically searched, the following points being noted :—

1. Cellularity.

   This was noted according to impression as low, normal or hypercellular. The classification corresponded almost exactly with the total counts recorded as under 20,000 ; 20,000–100,000 and over 100,000 respectively.

2. Plasma cell content.

   This was assessed on the general appearance including the finding of plasma cell nests, and on the differential marrow count.

3. The presence of abnormal cells in the marrow sample.

   As the films were examined microscopically cells that are not usually seen in marrow aspiration were encountered. These were then examined in further detail and an attempt made to define their exact nature. Some undoubtedly represented tumour cells while some were considered to be osteoclasts, osteoblasts or nests of degenerating plasma cells. The distinguishing features are considered later.

The two individual assessments were made entirely independently and the results then compared and the marrow classified into one of three groups namely:—

1. *Positive.*—The marrow contained cells considered to be definitely malignant.

2. *Suspicious.*—There was a definite plasmacytosis in the absence of any other cause with usually a hyperplasia of the other marrow elements. A few cases were included in this classification in which atypical cells were found but because they occurred in small numbers or singly could not be definitely classed as malignant cells. Such marrows, however, usually showed a plasmacytosis and would be classified as suspicious on that criteria as well.

3. *Negative.*—No abnormal cells and no abnormal bone marrow reactions were detected.

Only rarely has disagreement existed between the authors as to the classification and under these circumstances a compromise was reached as to the one recorded or if there was considerable doubt a repeat marrow examination has been made.

Marrow examinations have been made on 601 patients suffering from malignant disease. In addition to this, during this same time over 200 patients with diseases of the reticulo-endothelial system (e.g. leukaemia, Hodgkin's disease) had a marrow examination, but these patients are omitted entirely from discussion in this paper since marrow involvement may occur early in these diseases and the changes are already well recognised. Sarcomata were also excluded as the grade of malignancy is difficult to interpret. In addition, as a control, we have re-examined over 1,000 marrow punctures made during the past few years on patients with anaemia, on normal and abnormal infants (Gairdner, Marks and Roscoe, 1952) and many other generalised diseases including ankylosing spondylitis (Pillers and Marks, 1956). Among this control series no marrow which we would classify as positive was found.

*Clinical assessment of the patient*

The clinical stage of advancement of the malignant disease was recorded as early, intermediate or advanced according to the following criteria :—

1. *Early.*—Those patients with only apparently localised disease and with no detecable spread beyond the regional lymph nodes. This includes cases with mobile enlarged lymph nodes but excludes fixed nodes.

2. *Intermediate.*—Those patients showing clinical evidence of spread beyond (1) but no clinical evidence of involvement of the liver or clinical or radiological evidence of bone metastases.

3. *Advanced.*—Those patients in whom there is radiological evidence or clinical evidence of widespread metastases particularly in the bones or liver.

In addition to this assessment of the stage of advancement of the malignant disease patients were given a general medical examination. Most of the patients investigated were in good general health and in this present investigation much effort has been made to concentrate on the early cases rather merely examining patients already showing obvious evidence of widespread malignant disease.

---

EXPLANATION OF PLATES.

FIG. 1*a*.—Low power ( × 140) and high power ( × 700) view of cluster of malignant cells.
Note :—1. Grouped cells with syncytium (*a*) of lacy cytoplasm and indistinct borders. 2. Nuclei large, hyperchromatic, stippled chromatin and nucleoli (*b*).

FIG. 1*b*.—Group of osteoblasts ( × 550).
Note :—1. Eccentric nuclei. 2. Indistinct margin of cytoplasm (brilliant blue). 3. Archoplasm (*a*) may be at distance from nucleus. 4. Nucleoli (*b*).

FIG. 1*c*.—Osteoclast ( × 550).
Note :—1. Syncytial granulated cytoplasm (*a*). 2. Large round disconnected nuclei with stippled chromatin. 3. Nucleoli (*b*).

FIG. 1*d*.—Plasma cell " group " ( × 550).
Note :—1. Eccentric nucleus with dense chromatin. 2. Cytoplasm (dense blue) shows clear margin. 3. Perinuclear archoplasm (*a*).

1a

1b

1c

1d

The clinical staging we have used corresponds in the case of carcinoma of the cervix uteri to the following Stockholm staging. (Stockholm Report, 1951).

| | |
|---|---|
| Early | = I + II |
| Intermediate | = III |
| Advanced | = IV |

The survival times were calculated from date of marrow examination to the present time or to the date of death.

### RESULTS

The present series may be divided into five main groups for consideration of results and the discussion, namely carcinomata of the breast, bronchus, cervix and ovary and a heterogenous group in which the individual types of tumours were too few to warrant separate detailed consideration. Details of the number and type of the cases that have been punctured and a summary of the marrow results are shown in Table I.

TABLE I.—*Details of the site of the primary tumour and the marrow results*

| Site of neoplasm. | No. of patients studied. | Marrow results. | | |
|---|---|---|---|---|
| | | Positive. | Suspicious. | Negative. |
| Breast | 190 | 12 | 42 | 136 |
| Lung | 125 | 14 | 47 | 64 |
| Cervix | 61 | 1 | 23 | 37 |
| Ovary | 30 | 2 | 14 | 14 |
| Upper resp. tract | 29 | 0 | 9 | 20 |
| Alimentary canal | 28 | 1 | 11 | 16 |
| Male genital tract | 20 | 0 | 2 | 18 |
| Female genital tract | 15 | 0 | 5 | 10 |
| Urinary tract | 17 | 0 | 7 | 10 |
| Skin | 20 | 1 | 4 | 15 |
| Melanoma (all sites) | 10 | 0 | 2 | 8 |
| Endocrine system | 11 | 0 | 4 | 7 |
| Miscellaneous | 19 | 0 | 3 | 16 |
| Carcinomatosis (unknown primary) | 26 | 6 | 7 | 13 |
| Totals | 601 | 37 | 180 | 384 |

*Results in patients wth carcinoma of the breast*

Excluding male patients and patients with a second primary neoplasm 190 patients with primary carcinoma of the breast have been examined.

The marrow results and survival times to date in this series are given in Fig. 2.

The survival times calculated to date for these patients show no obvious difference between the negative, suspicious or positive marrows although such results cannot be of great value at present in assessing the prognosis since 129 of the patients are still alive. The figures, however, may be considered in a different way, if evidence of progression of disease is taken as a criteria in each of the marrow groups.

Among the 136 patients with negative marrow 54 patients (40 per cent) are either dead or show evidence of disease progression. In the suspicious marrow group 26 patients (approximately 62 per cent) have progressed since the marrow examination was made, while in the 12 patients with positive marrows 9 (75 per cent) of the patients show progression of the disease and 7 of these are dead.

Thus the results suggest that a further follow-up may result in a gradual difference in prognosis appearing between the three groups of patients since, in the negative group, there are far more patients who are now clinically and radiologically normal.

FIG. 2.—Results in 190 patients with carcinoma of the breast.

*Results in patients with carcinoma of the bronchus*

One hundred and twenty-five patients suffering from primary carcinoma of the bronchus have been examined. The diagnosis of carcinoma of the bronchus has been confirmed in these patients by thoracotomy, by biopsy, by bronchoscopy or in a very few cases only by the finding of malignant cells in pleural effusion or sputum.

The marrow results in this series and the survival times are given in Fig. 3.

FIG. 3.—Results in 125 patients with carcinoma of the bronchus.

The division of these patients into stages is much more difficult than with other carcinomata. Early patients are those who show no extrathoracic spread and no involvement of the ribs and who on bronchoscopic evidence were considered suitable for surgery. Many of these patients subsequently underwent thoracotomy. The advanced cases were those who were, at the time of marrow examination, known to have liver or bone involvement, while the intermediate group showed some extrathoracic spread but no clinical or radiological evidence of spread to the liver or bones.

The average survival times for the patients with negative marrows was seven months, for the suspicious marrows six months, and for the positive marrows five months. Since only nine of the patients are still alive with no evidence of spread of the disease these survival times are not likely to be greatly modified except for a possible slight prolongation of the group with negative marrows relative to the others.

*Results in patients with carcinoma of the cervix uteri*

The results of the marrow findings and the survival times for 61 patients with carcinoma of the cervix uteri who have been investigated are given in Fig. 4. Patients with negative marrow findings had an average survival time of sixteen

FIG. 4.—Results in 61 patients with carcinoma of the cervix.

months, those with suspicious marrows an average of eight months, while the patient with a positive marrow, even though classed as an intermediate case clinically died two months after the finding of a positive marrow. There is a statistically significant difference between the survivals in the negative and suspicious group. (Students t-test — $n = 32$ ; $t = 3{\cdot}36$ ; $P = 0{\cdot}002$ ; inclusion of the one positive marrow makes no difference to this significance.)

Among the patients with negative marrows 23 (63 per cent) are alive with no evidence of spread, while in the suspicious group only 3 (13 per cent) are still alive and well. Thus the difference between the negative and suspicious marrows should become considerably greater with a longer follow up.

The only positive marrow in this series showed no radiological evidence of spread and was, at the time of the marrow examination, in fairly good general condition. She died however two months later with clinical evidence of widespread disease.

*Results in patients with tumours of the ovary*

Thirty patients with ovarian tumours have been studied and the results are shown in Fig. 5. Classification into clinical stages has been done entirely on the findings at operation, early cases showed no evidence of spread outside the ovary and were operable. The intermediate group showed local extension and were inoperable as far as radical surgery was concerned, while advanced cases showed liver and bone secondaries.

The average survival time in the patients with negative marrows was sixteen months, with suspicious marrows eleven months, and in two patients with positive marrows two months.

Both the positive marrow patients are dead, and only 2 (14 per cent) of the patients with suspicious marrows showed no evidence of further spread of disease.

FIG. 5.—Results in 30 patients with ovarian tumours.

Among the patients with negative marrows 4 (28 per cent) are still alive and well. Thus in this group too, difference in survival times is likely to become more marked relative to the marrow classification rather than less marked. (Students t for all negative against positive and suspicious n = 28 ; t = 2·32 ; P = 0·27 and is significant at the 5 per cent level.)

*Results in a group of patients with carcinoma of various sites and types*

One hundred and ninety-five patients suffering from primary neoplasms of sites other than those already mentioned have been investigated. The diagnosis and the marrow findings are given in Table I. In a mixed group of this sort with diseases having varying natural histories follow up data would be valueless. Positive marrow aspirations were found only in two patients in whom the site of the primary growth was known (stomach and skin), but six patients with carcinomatosis with an unknown primary tumour have yielded a positive aspiration. Suspicious marrows were found only in 3 patients.

*The marrows eosinophil count in patients with malignant disease*

A total eosinophil percentage in the marrow has been calculated for each puncture. Table II gives details of the patients of the four main groups who showed a marrow eosinophilia (over 7·5 per cent). Eighteen out of 406 patients show a marrow eosinophilia but there is no apparent correlation in the clinical status or the follow up of these patients.

No eosinophilia was found among the patients with positive marrows and the incidence of eosinophilia among the patients with suspicious marrows was not more than would be expected by chance if the frequency of each of these conditions is considered.

TABLE II.—*Clinical staging, marrow result, and actual and calculated follow-up on patients showing a marrow eosinophilia*

| Diagnosis. | Case No. | Clinical status. | Marrow result. | Follow-up. | Expected follow-up. |
|---|---|---|---|---|---|
| Carcinoma, breast | 33 | E | — ve | 33 spread | 21 |
| | 77 | E | — ve | 7 dead* | 21 |
| | 79 | A | sus | 7 dead | 5 |
| Carcinoma, bronchus | 2 | I | sus | 3 dead* | 5 |
| | 10 | I | sus | 10 dead | 5 |
| | 11 | E | sus | 11 dead | 9 |
| | 26 | I | — ve | 2 dead* | 7 |
| | 28 | E | sus | 4 dead* | 9 |
| | 60 | I | — ve | 2 dead* | 7 |
| | 92 | I | — ve | 11 dead | 7 |
| Carcinoma, cervix | 7 | I | — ve | 38 a. & w. | 12 |
| | 17 | E | — ve | 29 „ „ „ | 20 |
| | 20 | E | sus | 3 dead* | 11 |
| | 33 | I | — ve | 20 a. & w. | 12 |
| | 40 | I | sus | 2 dead* | 8 |
| Ovarian tumours | 1 | I | sus | 12 dead | 12 |
| | 4 | I | — ve | 18 dead | 14 |
| | 12 | I | — ve | 12 dead* | 14 |

* Patient with a poorer prognosis than expected.

### DISCUSSION

### *The identification of malignant cells in the marrow*

The identification of malignant cells in the marrow is often a matter of considerable difficulty, and requires knowledge of the normal marrow cells under a variety of conditions.

Since the primary tumours differ so much in their morphology it is only to be expected that the nodules in the marrow would show a similar considerable variation in cytology. Thus it is only possible to give broad generalisations of the distinguishing features. These features can be summarised as :—

1. The general sturcture and staining qualities of the cells are at variance with those of the normal cells of the marrow, i.e. the cells are foreign to the normal marrow elements.

2. The cells are usually arranged in a group of cluster and have a tendency to form a syncytium.

3. There is a great variation in the cell size, but the cells are usually large—10 to 30$\mu$ in diameter.

4. The cytoplasm may be abundant or scanty. When abundant it is often fragile, lacy or vacuolated with indistinct borders and may even be absent. It may be slightly or deeply basophilic in staining.

5. The nucleus is usually large and hyperchromatic with stippled nuclear chromatin.

6. The nucleoli are usually prominent, usually 1–3 in number and may be disproportionately large in relation to the nucleus. As described by Stoger (1941) the smaller cells may have no nucleoli, but the finding of these small malignant cells with no nucleoli is extremely rare and the authors feel that considerable care should be exercised in identifying these as true tumour cells. The majority of such cells are probably plasma cells.

7. Many other workers describe mitotic figures as being numerous, but in our experience they appear to be numerous only in those cases in which the marrow is entirely replaced by sheets of malignant cells.

At the present stage of the investigation it is not considered possible to determine the site of the primary tumour from the cytological characteristics of the cells found at bone marrow puncture. Rohr and Hegglin (1936) suggested that small cells occurring in groups might indicate a secondary from a carcinoma of the bronchus while large cells sometimes occurring singly could suggest the prostate or stomach as primary. Amy and Jaimet (1953) reported similar findings. Rohr's figures are however on the basis of 7 positive aspirations in carcinoma of the bronchus and in intestinal carcinoma. In the present work we have been unable to confirm their conclusions in carcinoma of the bronchus for out of the 14 positive aspirations that we report only 2 showed clusters of small cells. There are certain other cells which may easily be confused with malignant cells in the marrow. Some of these have undoubtedly confused other authors to judge from the photomicrographs that they reproduce and have caused us considerable worry at times in arriving at a firm conclusion.

Fig. 1*a* shows a typical cluster of malignant cells.

*Osteoblasts and osteoclasts*

These cells would seem to produce more confusion than the rest. They were described by Esser in 1945 and were stated by her to occur much more commonly in children's marrows than adult. Although these cells are normal in bone biopsies they are seen only rarely in aspiration samples of marrow. They may occur either singly or more commonly in our experience in small clusters.

The osteoblasts closely resemble plasma cells with their eccentric nuclei and brilliant blue cytoplasm, but are much larger in size—20–30$\mu$. They may show azurophilic granules, and the margin is usually indistinct. The pale round area of archoplasm is some distance from the nucleus whereas it is adjacent to the nucleus in plasma cells. There are usually one or two nucleoli.

The osteoclasts are multinucleate cells with big round disconnected nuclei, and granular cytoplasm which shows some resemblance to the cytoplasm of megakaryocytes.

These cells have been stated (Tischendorf and Heckner, 1950) to be present in the marrow only under certain pathological conditions in adults but we have seen them both singly and in clumps not only in carcinoma, but also in Hodgkin's disease, spondylitis and other non-malignant conditions. As far as we can determine they have no especial significance relative to the conditions, but may provide diagnostic difficulty.

Fig. 1*b* and 1*c* show typical osteoblasts and osteoclasts in sternal marrow aspiration.

*Plasma cell nests and degenerating plasma cells*

Degenerating plasma cells may occur either singly or in clumps, and are larger than most other normal marrow cells. The nucleus may show pyknosis with early fragmentation and occasionally an intra-nuclear vacuole which may give the appearance of a false nucleolus. The cytoplasm is plentiful and usually faintly staining, while the cell outline is often indistinct. These cells are described by Hayhoe and Smith (1951) and occur in many conditions other than malignant disease.

Plasma cells commonly occur singly in the marrow but where there is a plasmacytosis they may also be found in nests. Frequently these nests contain a multinucleate plasma cell in addition to several with single nuclei, and the first impression is often of a syncytium. The dense blue cytoplasm, the dense chromatin in the nucleus with no nucleoli and the perinuclear archoplasm, distinguish these nests from a cluster of malignant cells.

A typical plasma cell group is shown in Fig. 1*d*.

*The incidence of positive aspirations*

Among the present series there has been an incidence of 6·2 per cent positive aspirations (37 out of 601 patients) or 7 per cent in the four main groups considered (29 out of 406 patients). This at first sight compares very unfavourably with the results of previous workers (Table III), in patients with similar diagnoses. Thus overall they found approximately 32 per cent of the patients had positive marrow findings. This is to a very considerable extent due to the different selection of patients. Among the patients with positive aspirations in the four main groups of this present series 18 or 62 per cent were found in patients with no radiological evidence of spread of disease. In the patients investigated by other workers on the other hand only 10 per cent of the positive aspirations were found among patients with no radiological evidence of spread. Previous workers have in fact almost entirely confined their attentions to patients with advanced disease. The present series on the other hand deliberately concentrated on the less advanced cases.

TABLE III.—*Comparison of the positive marrow aspirations in the present series with those reported by previous workers in patients with primary tumours of similar sites*

| Author. | Breast. | Lung. | Cervix. | Ovary. | Total. |
|---|---|---|---|---|---|
| Vogel, Erf and Rosenthal 1937 | 2 (2) | 0 (5) | 0 (1) | — | 2 (8) |
| Kryeberg and Poppe, 1940 | 3 (26) | 0 (1) | 0 (1) | — | 3 (28) |
| Selberg, 1942 | 3 (6) | 4 (12) | . | — | 7 (18) |
| Franke, 1942 | 2 (11) | 5 (22) | 2 (5) | 0 (2) | 9 (40) |
| Gormsen, 1942 | 19 (98) | 2 (7) | — | — | 21 (105) |
| Weisberger and Heinle, 1948 | 2 (10) | 2 (8) | 0 (2) | — | 4 (20) |
| Lanier, 1949 | 2 (5) | 0 (2) | 0 (2) | — | 2 (9) |
| Jonsson and Rundles, 1951 | 8 (14) | 6 (14) | — | — | 14 (28) |
| Rubinstein and Smellin, 1951 | 21 (35) | 4 (10) | — | — | 25 (45) |
| Rubinstein and Smellin, 1952 | 20 (32) | 5 (10) | — | — | 25 (47) |
| Total | 82 (239) | 28 (96) | 2 (11) | 0 (2) | 112 (348) |
| Present series | 12 (190) | 14 (125) | 1 (61) | 2 (30) | 29 (406) |
| | 4* | 12* | 1* | 1* | 18* |

* Denotes the number of patients showing positive marrow aspiration and no X-ray evidence of bone metastases.

*Sternal aspiration as a method for the detection of tumour cells*

In order to find tumour cells in the maximum number of patients one should puncture the bone that shows radiological evidence of spread. We have, on occasions, taken radiographs of various bones including the sternum to find a suitable site for puncture but such radiographic examinations are costly and time consuming and are not feasible in many units undertaking large numbers of routine examinations. In addition it has already been shown that positive aspirations may be found where there is no radiological evidence of disease.

Bone marrow aspiration can be performed in the sternum, the iliac crest and the vertebrae. There is evidence both from autopsy examinations (Selberg, 1942) and from patients with multiple examinations (Clifton, Philip and Fowler, 1952) that sternal marrow aspiration yields a slightly higher number of positive results than either iliac crest or vertebral spine examinations where previous radiographic investigation is not undertaken. The finding of cells in the sternal marrow cannot be ascribed to direct extension in carcinoma of the breast and bronchus.

Thus where it is impossible to determine the site of secondaries by radiographic examination or where there is no evidence of spread, examination of the sternum is probably the method of choice and is the one that we have adopted.

*The significance of a marrow plasmacytosis*

An increase of plasma cells in the marrow has been reported in a variety of conditions both physiological and pathological. Among these may be mentioned rheumatoid arthritis (Hayhoe and Smith, 1951). chronic infections, measles, cirrhosis, and Hodgkin's disease ; for review of cause of marrow plasmacytosis *vide* Fadem and McBirnie (1950).

Such an increase has however been reported to occur in malignant disease and in such cases may indicate the presence of a generalised spread. Stoger (1941) examined the marrows of 110 patients with malignant disease and found that in 84 per cent of his tumour free biopsies a plasma cell reaction from 1·1–16 per cent plasma cells was present. Morel (1947) suggested that a plasmacytosis greater than 4 per cent suggested the presence of a widespread neoplasm, while Lanier (1949) in her series of 32 cases found numerous clumps of plasma cells. Amy and Jaimet (1953) similarly found an increase of plasma cells from 2 to 13 per cent.

In view of the numerous conditions that can give rise to a plasmacytosis this reaction can in no way be considered specific for malignancy. Such a reaction should be considered as evidence of some generalised body disturbance and a cause sought. In the absence of other cause in a patient with an already proven malignancy it is possible that the presence of this neoplasm may be the exciting factor.

In the present series those patients with a plasmacytosis have been fully investigated in an attempt to discover any other possible cause. When none has been found they have been included in the classification of patients with a suspicious marrow. The significance of this plasmacytosis in relation to the prognosis of patients with malignant disease is considered in a subsequent section.

*The significance of a bone marrow eosinophilia*

A few previous workers have noted an eosinophilia in the bone marrow of patients with neoplastic disease. Morel (1947) felt an increase of eosinophils of over 5 per cent was significant of neoplasm where no other cause could be found.

Lanier (1949) found an increase in 44 per cent of her patients but cited Nordensen (1935) as stating that it was an infrequent occurrence in his series of 17 patients.

In the present series 18 out of the 406 patients analysed showed an eosinophilia (over 7·5 per cent mature eosinophil and precursors). There is at present no apparent correlation between the clinical status and the follow up of these patients. No eosinophilia was detected in the 37 patients with positive bone marrow aspirations. Eight of the 18 patients with eosinophilia were classified by other criteria as suspicious.

Among the patients with a marrow classification of suspicious and an eosinophilia 4 (50 per cent) had a prognosis poorer than the average expected survival time, among those with eosinophilia and negative marrows 4 (40 per cent) had a prognosis poorer than the average expected survival time.

Thus at the present time there is little evidence that eosinophilia can be considered to signify poor prognosis in malignant disease.

*The relationship between the marrow classification and the clinical follow up*

The four main groups considered have an entirely different natural history and therefore the results of the follow up study must be considered separately for each type of primary neoplasm.

In carcinoma of the bronchus patients showing a positive marrow have an average survival time of five months, those with a suspicious marrow six months and those with a negative marrow seven months with a greater percentage of the patients still alive and well who had a negative marrow examination. Thus the prognosis when a positive marrow was found seems to be rather shorter than with either a suspicious or a negative marrow while a suspicious marrow gives a slightly poorer prognosis than the negative marrow. Differences in the average survival times however are short.

In carcinoma of the cervix average survival times are significantly longer with a negative marrow than with either a suspicious marrow or a positive marrow, and the patient with a positive marrow showed an extremely short survival time. These differences are likely to become more pronounced for the majority of the patients with a negative marrow are still alive and well.

In ovarian tumours two patients with a positive marrow showed a very short survival time while those patients who had a negative marrow had a slightly better prognosis than those with a suspicious marrow.

In carcinoma of the breast no differences can be found so far in the series examined between the average survival times of patients with negative, suspicious and positive marrows but in carcinoma of the breast the overall survival time is long and the majority of patients are still alive and well. If evidence of spread of the disease is considered, however, more patients with positive and suspicious marrows show evidence of spread. A longer follow up however will be necessary before clear-cut results can be found for carcinoma of the breast.

Considering each of these groups there is evidence to suggest that correlation exists between the marrow findings and the clinical follow up. Marrow puncture can give evidence of spread of the disease but although this would on the average signify a poor prognosis it does not of course necessarily signify a very early death and should be used as a further indication of general spread.

The finding of a suspicious marrow on the other hand cannot be considered as pathognomonic of malignant spread for many other conditions can give these changes. A suspicious marrow however must not be dismissed until a cause has been found. Should no other cause be determined, there is evidence from this present study to suggest that tumour spread may exist with a consequent poor prognosis to the patient.

### CONCLUSIONS

*The value of sternal puncture in the treatment of carcinona*

The present results in this series suggest that the following firm conclusions may be drawn.

1. Sternal puncture has a definite value in assessing the prognosis in both carcinoma of the cervix and carcinoma of the ovary but has little value in determining the prognosis in carcinoma of the bronchus. In carcinoma of the breast the natural history of the disease is so long that no definite conclusions can be drawn at present as to the value of sternal puncture in assessing prognosis.

2. Sternal puncture has a definite place in assessing the type of treatment that should be used.

(*a*). Carcinoma of the bronchus.

Where there is evidence of spread as determined by a positive marrow aspiration major surgery should be avoided except as a purely palliative measure.

(*b*) Carcinoma of the ovary.

Here a decision must be made between palliative and radical X-ray therapy. With a negative marrow examination there is strong evidence from this series that no spread exists and under these circumstances radical therapy in the maximum tolerated dose is indicated.

(*c*) Carcinoma of the cervix.

Those patients who show a suspicious or positive marrow and fail to respond locally to primary radiotherapy have not benefited greatly from Wertheim's radical abdominal hysterectomy.

3. It is possible by sternal marrow examination to detect spread of tumour earlier than with X-rays but the method is extremely time-consuming (about three hours per patient) and requires considerable experience of normal and abnormal bone marrows. It consequently will probably have no place in the routine radiotherapeutic department.

4. Sternal marrow may provide cytological material for diagnosis but at the present moment only of the presence of malignancy. With further experience the site of the primary tumour may possibly be diagnosed.

5. The finding of a suspicious marrow should be used as a guide to the presence of organic disease, and a suspicious marrow should never be dismissed until the cause is determined.

### SUMMARY

The results are reported on sternal marrow examination of 601 patients suffering from carcinoma. The majority of these patients were clinically well and at an early stage of the disease. Four hundred and six patients with carcinoma of the breast, bronchus, cervix uteri and ovary are considered in detail.

The follow-up has varied from six months to over 4 years.

Thirty-seven patients showed tumour cells in the marrow, while 180 showed a marrow plasmacytosis. Evidence is presented that prognosis is worst with tumour cells in the marrow, but that in carcinoma of the bronchus, cervix uteri and ovary, a marrow plasmacytosis is associated with a poorer average survival than patients showing no marrow plasmacytosis.

It is suggested that sternal marrow examination has a place in assessing the type of treatment and in providing cytological material for diagnosis of malignancy.

Our thanks are due to Mr. B. McN. Truscott and Mr. C. Parish for permission to publish cases under their care and to Dr. Herman Lisco for his advice.

Part of this work was submitted as a thesis for an M.D. Camb. One of us (E.M.K.P.) has been supported by grants from the Damon Runyon Research Fund and the Medical Research Council.

### REFERENCES

AMY, H. E. AND JAIMET, C. H.—(1953) *Canad. med. Ass. J.*, **69**, 424.
CLIFTON, J. A., PHILLIP, R. J. AND FOWLER, W. M.—(1952) *Amer. J. med. Sci.*, **244**, 121.
ESSER, M.—(1945) *Helv. med. acta*, **12**, 265.
FADEM, R. S. AND MCBIRNIE, J. E.—(1950) *Blood*, **5**, 191.
FRANKE, E.—(1942) *Z. klin. Med.*, **140**, 622.
GAIRDNER, D., MARKS, J. AND ROSCOE, J. D.—(1952) *Arch. Dis. Childh.*, **27**, 128.
GORMSEN, H.—(1942) *Nord. med.*, **14**, 1320.
HAYHOE, F. G. J. AND SMITH, D. R.—(1951) *J. clin. Path.*, **4**, 47.
JONSSON, U. AND RUNDLES, R. W.—(1951) *Blood*, **6**, 16.
KREYBERG, L. AND POPPE, E.—(1940) *Lancet*, i, 593.
LANIER, P. F. (1949) *Arch. intern. Med.*, **84**, 891.
LEITNER, S. J.—(1949) ‘ Bone marrow Biopsy.” London (Grune & Stratton), p. 365.
MOREL, P.—(1947) *J. Med. Lyon*, **28**, 477.
NORDENSEN, N. G.—(1935) ‘ Studies on Bone Marrow from Sternal Puncture.’ Stockholm (Börtzells).
PILLERS, E. M.K.—(1955) M.D. Thesis, Camb. Table X.
*Idem* AND MARKS, J.—(1956) *Lancet*, i, 722.
REICH, C.—(1935) *Amer. J. med. Sci.*, **189**, 515.
ROHR, K. AND HEGGLIN, R.—(1936) *Dtsch. Arch. klin. Med.*, **170**, 61.
RUBINSTEIN, M. A. AND SMELLIN, A.—(1951) *Acta haemat.*, **5**, 292.—(1952) *Arch. intern. Med.*, **89**, 909.
SELBERG, W.—(1943) *Dtsch. Arch. klin. Med.*, **190**, 380.
STOCKHOLM REPORT—(1951) Annual Report of the Result of Treatment in Carcinoma of the Uterus. Ed. Heyman. Nordstedt. Stockholm, 1951.
STOGER, R.—(1941) *Dtsch. med. Wschr.*, **67**, 1389.
TISCHENDORF, W. AND HECKNER, F.—(1950) *Klin. Wschr.*, **28**, 21.
VOGEL, P., ERF, L. A. AND ROSENTHAL, N. (1937) *Amer. J. clin. Path.*, **7**, 498.
WEISBERGER, A. S. AND HEINLE, R. W.—(1948) *Amer. J. med. Sci.*, **215**, 170.